# Integrative Genome-Wide DNA Methylome and Transcriptome Analysis of Ovaries from Hu Sheep with High and Low Prolific

**DOI:** 10.3389/fcell.2022.820558

**Published:** 2022-02-03

**Authors:** Xiaolei Yao, Fengzhe Li, Zongyou Wei, M. A. EI-Samahy, Xu Feng, Fan Yang, Feng Wang

**Affiliations:** ^1^ Hu Sheep Academy, Nanjing Agricultural University, Nanjing, China; ^2^ Jiangsu Livestock Embryo Engineering Laboratory, Nanjing Agricultural University, Nanjing, China; ^3^ Taicang Agricultural and Rural Science and Technology Service Center, and Graduate Workstation, Taicang, China; ^4^ Animal Production Research Institute, ARC, Ministry of Agriculture, Giza, Egypt

**Keywords:** DNA methylation, gene expression, ovary, prolificacy, Hu sheep

## Abstract

DNA methylation plays an important role in biological processes by affecting gene expression. However, how DNA methylation regulates phenotypic variation in Hu sheep remains unclear. Therefore, we generated genome-wide DNA methylation and transcriptomic profiles in the ovaries of Hu sheep with different prolificacies and genotypes (FecBB and FecB+). Results showed that ovary DNA methylome and transcriptome were significantly different between high prolificacy and low prolificacy Hu sheep. Comparative methylome analyses identified 10,644, 9,594, and 12,214 differentially methylated regions and 87, 1,121, and 2,375 genes, respectively, showing differential expression levels in three different comparison groups. Female reproduction-associated differentially methylated regions-related genes and differentially expressed genes were enriched, thereby the respective interaction networks were constructed. Furthermore, systematical integrative analyses revealed a negative correlation between DNA methylation around the transcriptional start site and gene expression levels, which was confirmed by testing the expression of integrin β2 subunit (ITGB2) and lysosome-associated protein transmembrane-4 beta (LAPTM4B) *in vivo* and *in vitro*. These findings demonstrated that DNA methylation influences the propensity for prolificacy by affecting gene expression in the ovaries, which may contribute to a greater understanding of the epigenome and transcriptome that will be useful for animal breeding.

## Introduction

Litter size is one of most important traits that determines the fecundity and reproductive efficiency of sheep bred for meat. In China, most sheep species are monotocous and seasonal estrus, although a few are polytocous. Compared with other sheep, Hu sheep is an excellent local breed in China, which is known for its high prolificacy and year-round estrus (Yue, 1996). Therefore, determining the molecular mechanisms associated with fecundity will help accelerate the breeding process of sheep with high prolificacy. Although the existing genetic studies have identified several genes with sheep fecundity, including GDF9, BMP15, and BMPR1B, the underlying genetic mechanisms remain largely unexplored (Chu et al., 2007; Polley et al., 2010; Farhadi et al., 2013; Wang et al., 2015). FecB (mutation in BMPR1B) is one of the key genes associated with sheep prolificacy (Wang et al., 2015). Moreover, evidences revealed that sheep with the homozygous mutation (FecBB; BB) had the greater ovulation rates than those with the heterozygous mutation (FecB+; B+) or the wild-type genotype (++) (Fabre et al., 2006; Gootwine et al., 2008). Thus, Hu sheep with different prolificacies and genotypes (BB and B+) were selected as experimental subjects.

It is widely thought that ovarian dysfunction leads to infertility in mammals. One of the main functions of the ovaries is to produce mature oocytes and secrete reproductive hormones that are involved in the follicular development and ovulation. Therefore, studying the differences in regulatory mechanisms in the ovaries of sheep with different prolificacies may reveal the mechanisms behind the genetic regulation of little size traits.

DNA methylation is an epigenetic modification that regulates gene expression and serves as a key regulator of development (Fan et al., 2020; Yang et al., 2021), differentiation (Khavari et al., 2010), and other processes (Miyakuni et al., 2021). Recent studies have shifted their focus toward how DNA methylation regulates pubertal onset (Lomniczi et al., 2015; Yang et al., 2016; Yuan et al., 2019), estrus (An et al., 2018; Zhou et al., 2018), ovary (Wang et al., 2014; Yu et al., 2015; Sagvekar et al., 2019), oocyte (Mattern et al., 2017; Wei et al., 2019), and embryo development (Salilew-Wondim et al., 2015; Canovas et al., 2017), and reproductive diseases (Vazquez-Martinez et al., 2019; Cao et al., 2021; Zhong et al., 2021). In-depth studies pertaining on epigenetic modifications, such as DNA methylation, are facilitating improvement in animal breeding (Gonzalez-Recio et al., 2015). Several studies have reported the genome-wide methylation profiles associated with milk production-related phenotypes in dairy cows (Singh et al., 2012), and disease resistance in crops (Tirnaz and Batley, 2019), as well as litter size in goats and pigs (Hwang et al., 2017; Kang et al., 2021). Meanwhile, Miao et al. (2017) recently reported the DNA methylation patterns in the ovaries of Dorset sheep and Small Tail Han sheep. These studies have demonstrated that DNA methylation plays an important role in regulating fecundity.

DNA methylation and gene expression profiles have been previously studied in the ovaries of Hu sheep with different prolificacies (the BB genotype only) to elucidate the regulatory mechanisms involved in Hu sheep fecundity (Zhang et al., 2017; Feng et al., 2018). As an extension of this study, here we aimed to systematically investigate both genome-wide DNA methylation and gene expression profiles in the ovaries of Hu sheep with different prolificacies (high prolificacy, HP; low prolificacy, LP) and genotypes (BB and B+) by performing whole-genome bisulfite sequencing (WGBS) and RNA-sequencing (RNA-seq). Furthermore, an integrated analysis of DNA methylation and transcriptome was performed to reveal whether and how DNA methylation mediate prolificacy phenotypic variations by affecting gene expression.

## Materials and Methods

### Animals and Sample Collection

We previously reported the details of the procedures for handling experimental animals and sample collection (Yao et al., 2021), which were approved by the Institutional Animal Ethics Committee of the Nanjing Agricultural University (SYXK 2011-0036). In brief, we firstly chose twenty non-pregnant ewes (2, 3 years old) based on their litter size numbers of three records, categorizing them as HP ewes (litter size = 3, *n* = 4) and LP ewes (litter size = 1, *n* = 16). Meanwhile, BMPR1B polymorphism genotyping of those ewes was detected as described previously (Yao et al., 2021). Finally, nine ewes were selected and divided into HPBB (*n* = 3), LPBB (*n* = 3) and LPB+ (*n* = 3) groups. All ewes were slaughtered during estrus and the ovarian samples were immediately collected and stored at −80°C for WGBS and RNA-seq.

### WGBS and RNA-seq

Genomic DNA and total RNA were extracted from ovarian tissue of the nine sheep using a Genomic DNA kit (Cat.#DP304-03,Tiangen, Beijing, China) and TRIzol reagent (Cat.# 15596-026; Invitrogen, Carlsbad, CA), respectively. The concentration and purity of isolated DNA and RNA were determined using NanDrop spectrophotometer (NanoDrop Technologies, Wilmington, DE, United States) and agarose gel electrophoresis. The preparation of nine genome-wide DNA methylation and transcriptome libraries and sequencing (WGBS and RNA-seq), respectively, were performed by Biomarker Technologies Corporation (Beijing, China). Notably, we previously reported the DNA methylation (Zhang et al., 2017) and mRNAs (Feng et al., 2018) profiles in ovaries of only HPBB and LPBB Hu sheep, and using the same described methods, we analyzed the WGBS and RNA-Seq data in this study, with some modifications. Briefly, the raw data were first filtered to remove low-quality reads, and the clean data were then aligned with the reference genome of sheep (Ovis aries v4.0).

Differential methylated regions (DMRs) were defined by the presence of at least three methylation sites in the region, and the difference in methylation levels was >0.2 (>0.3 for CG type) with *P*-value (Fisher’s) < 0.05. Differentially expressed (DE) mRNAs were identified with the false discovery rate (FDR) < 0.05 and absolute value of log_2_ (fold change, FC) >1. Primarily, DMGs were detected through mapping the DMRs to genes based on their genomic location. In this study, we defined the genomic region from −3000 bp to the transcription start site (TSS) as the promoter region and from the TSS to the transcriptional termination site (TTS) as the gene body region. To visualize the overlapping gene sets, we generated venn diagrams using Venn diagram online tool (http://bioinformatics.psb.ugent.be/webtools/Venn/).

### Integrated Analysis

The correlation between gene expression and methylation was calculated using only differentially expressed genes (DEGs) and DMR-related genes (DMGs). For the global methylation around TSS regions (±2000 bp) and gene expression correlation analysis, the genes was divided into four groups according to their expression level: hightest, lowest, medium-high and medium-low. T visualize the relationship between methylation and gene expression, heatmaps were generated using the R package (heatmap).

### Functional Enrichment Analysis

Gene Ontology (GO) enrichment analyses of DMGs and DEGs were implemented by the Wallenius non-central hyper-geometric distribution in the GOseq R packages (Young et al., 2010). Kyoto Encyclopedia of Genes and Genomes (KEGG) analysis of DMGs and DEGs were evaluated using KOBAS software (Mao et al., 2005).

### Interaction Network Construction

The interaction network of DMGs and DEGs associated with female reproduction was analyzed using the STRING database (http://string-db.org/) and visualized with Cytoscape software (V3.4.0).

### Bisulfite Sequencing PCR

For BSP analysis, genomic DNA of ovaries from each groups was modified with sodium bisulfite using an EZ DNA Methylation-Direct Kit (Cat.#D5020; Zymo Research, Irvine, CA, United States). The PCR products were purified using a gel extraction kit (Cat.#DC301-01; Vazyme, Nanjing, China), then ligated and cloned into the pMD19-T vector (Cat.#6013; Takara, Osaka, Japan). Subsequently, ten positive clones of each sample were randomly selected for DNA sequencing. Data were analyzed and visualized using BIQ Analyzer software. BSP primers are listed in Supplementary Table S1 and all operations were conducted according to the manufacturer’s instructions.

### Cell Culture and Treatments

Hu sheep granulosa cells (GCs) were isolated from healthy follicles (2–5 mm) and cultured as our previously described (Yao et al., 2021). Briefly, GCs were seeded into different plates (6-well: 1 × 10^6^ cells/well) in culture medium (Dulbecco’s modified Eagle’s medium/nutrient mixture F-12 supplemented with 10% fetal bovine serum, 2 mM L-glutamine, 100 IU/ml penicillin, and 100 μg/ml streptomycin) at 37°C and 5% CO_2_. When the cultured GCs attained 60–70% confluence, the culture medium was replaced with new medium containing various concentrations (0, 1, 2, 5, 10, and 20 μM) of 5-Aza-deoxycytidine (5-Aza; Cat.#a3656; Sigma-Aldrich, United States), as a DNA methyltransferase inhibitor, and incubated for 48 h.

ITGB2 (integrin β2 subunit) knockdown was achieved using siRNAs, which were synthesized by GenePharma (Shanghai, China) and the sequences listed in Supplementary Table S2. After cultured GCs to 60–70% confluence, siRNA-NC and siRNA-ITGB2 were transfected into GCs with or without 10 μM 5-Aza for 48 h. Subsequently, all treated cells were collected for further analysis. All reagents used in cell culture were purchased from Life Technologies (Pleasanton CA, United States).

### Immunohistochemistry

Immunohistochemistry was performed following our previously described method (Yao et al., 2017). Rabbit anti-LAPTM4B (lysosome-associated protein transmembrane-4 beta; Cat.#18895 -1-AP; ProteinTech, Rosemont, IL, United States) were used as primary antibody. For negative control, the primary antibody was replaced with Tris-buffered saline. Digital images were examined using a light microscope (Nikon, Tokyo, Japan).

### Quantitative Reverse Transcriptase PCR

For qRT-PCR analysis, cDNA was synthesized using reverse transcription reagent kit with gDNA wiper (Cat.#R323-01; Vazyme, Nanjing, China). Subsequently, qRT-PCR was performed on an ABI 7500 Real-Time PCR System (Applied BioSystems, Carlsbad, CA, United States) using SYBR Green Master Mix (Cat.#Q711-02; Vazyme, Nanjing, China). Relative mRNA expression levels were quantified using 2^–ΔΔCT^ method, with Glyceraldehyde 3-phosphate dehydrogenase (GAPDH) as the internal control. Primers were designed using the Primer 5.0 software and listed in Supplementary Table S3.

### Statistical Analysis

Data are presented as means ± SEM based on three independent experiments, with GraphPad Prism v7.0 (GraphPad Software, CA, United States). Statistical analyses were performed by the one-way analysis of variance followed by a post hoc test in the SPSS25.0 software package (Chicago, IL, United States). *P-values* < 0.05 were considered statistically significant.

## Results

### Overview of Genome-Wide DNA Methylation Profiling in the Ovaries of Hu Sheep

To determine the effects of DNA methylation on the phenotypic variation of prolificacy, WGBS analysis was performed in the ovaries of HPBB, LPBB and LPB + groups of Hu sheep. After data filtering, each sample had approximately 210 million clean reads. Of mapped reads, 74.26, 73.94, and 66.65% in the HPBB, LPBB and LPB + groups, respectively, were used for subsequent analysis, with an average of 3.58% of methylated cytosine sites (Supplementary Table S4).

**FIGURE 1:**
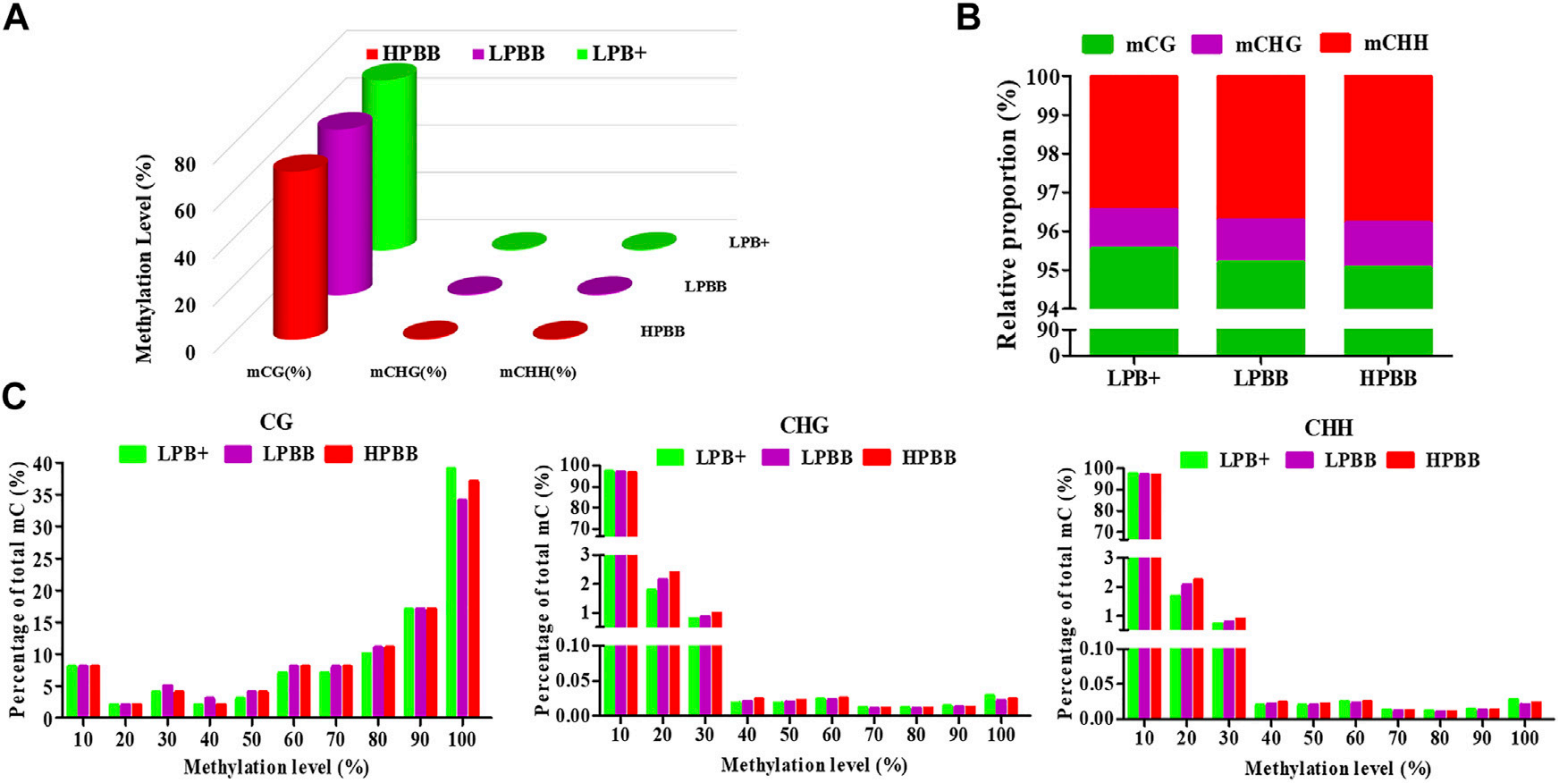
DNA methylation levels and distribution in ovaries of Hu sheep with different prolificacies. **(A)** Genomic methylation levels of CG, CHG, and CHH in different groups. **(B)** Relative average proportions of CG, CHG, and CHH methylation contexts in ovaries among different groups. **(C)** The average methylation percentage (y-axis) for different methylation levels (x-axis) of CG, CHG, and CHH methylation in different groups.

Global DNA methylation profiles indicated three contexts, CG, CHH, and CHG (where H is A, C, or T), existed in the ovaries of Hu sheep. The genomic methylation proportions of CG, CHG, and CHH contexts was respectively 72.23, 0.50 and 0.50% in the LPB + group, 69.60, 0.63 and 0.63% in the LPBB group, and 70.77, 0.70 and 0.66% in the HPBB group (Figure 1A). Among the three methylated contexts (CG, CHG and CHH), the methylated CG context represented 95.55, 95.20 and 95.06% in the LPB+, LPBB and HPBB groups, respectively (Figure 1B). In addition, the methylated level of CG context was mainly between 90 and 100%, whereas the methylated levels of CHG and CHH contexts apparently exhibited a more uniform distribution range from 10 to 30% (Figure 1C).

### Comparison of DMRs in the Ovaries of Hu Sheep with Different Prolificacies

**FIGURE 2:**
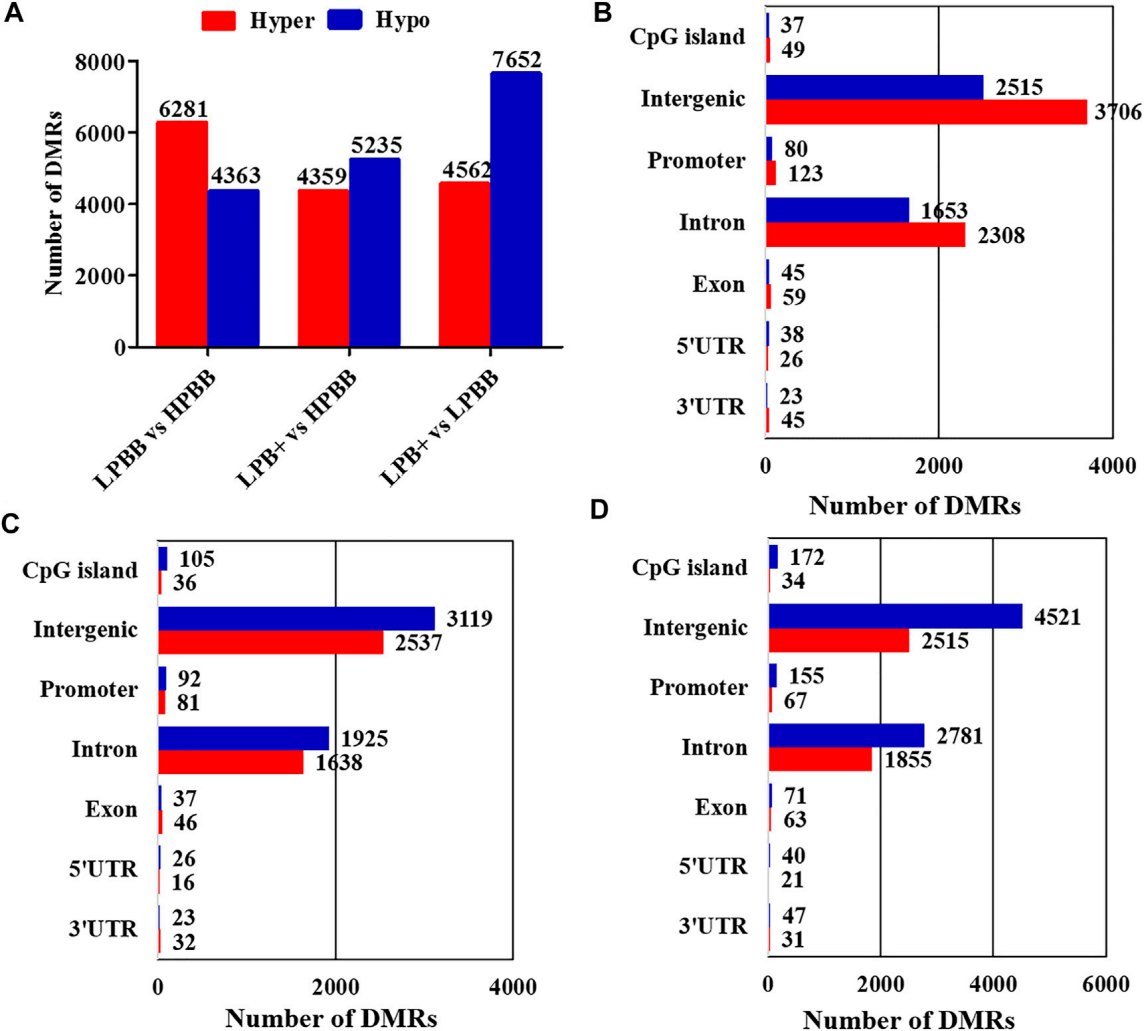
Identification of DMRs in different groups. **(A)** The number of DMRs (hyper- and hypo-methylated) in different comparison groups. **(B,C)** The distribution of DMRs in different genomic elements in the **(B)** LPBB vs. HPBB, **(C)** LPB + vs. HPBB, and **(D)** LPB + vs. LPBB groups.

The Model-Based Analysis of Bisulfite Sequencing package was used to identify DMRs and compare the DNA methylation profiles between the different groups. Overall, 10,644 DMRs (6,281 hyper-methylated and 4,363 hypo-methylated; 10,634 CG, 1 CHG and 9 CHH), 9,594 DMRs (4,359 hyper-methylated and 5,235 hypo-methylated; 9,583 CG, 2 CHG and 9 CHH) and 12,214 DMRs (4,562 hyper-methylated and 7,652 hypo-methylated; 12,199 CG, 1 CHG and 14 CHH) were identified in the LPBB vs. HPBB, LPB + vs. HPBB and LPB + vs. LPBB groups, respectively (Figure 2A and Supplementary Tables S5–S7). Moreover, the distribution of DMRs were mainly located at intergenic and intron regions (Figures 2B–D).

### Identification of DMGs and DEGs Among Different Comparison Groups

**FIGURE 3:**
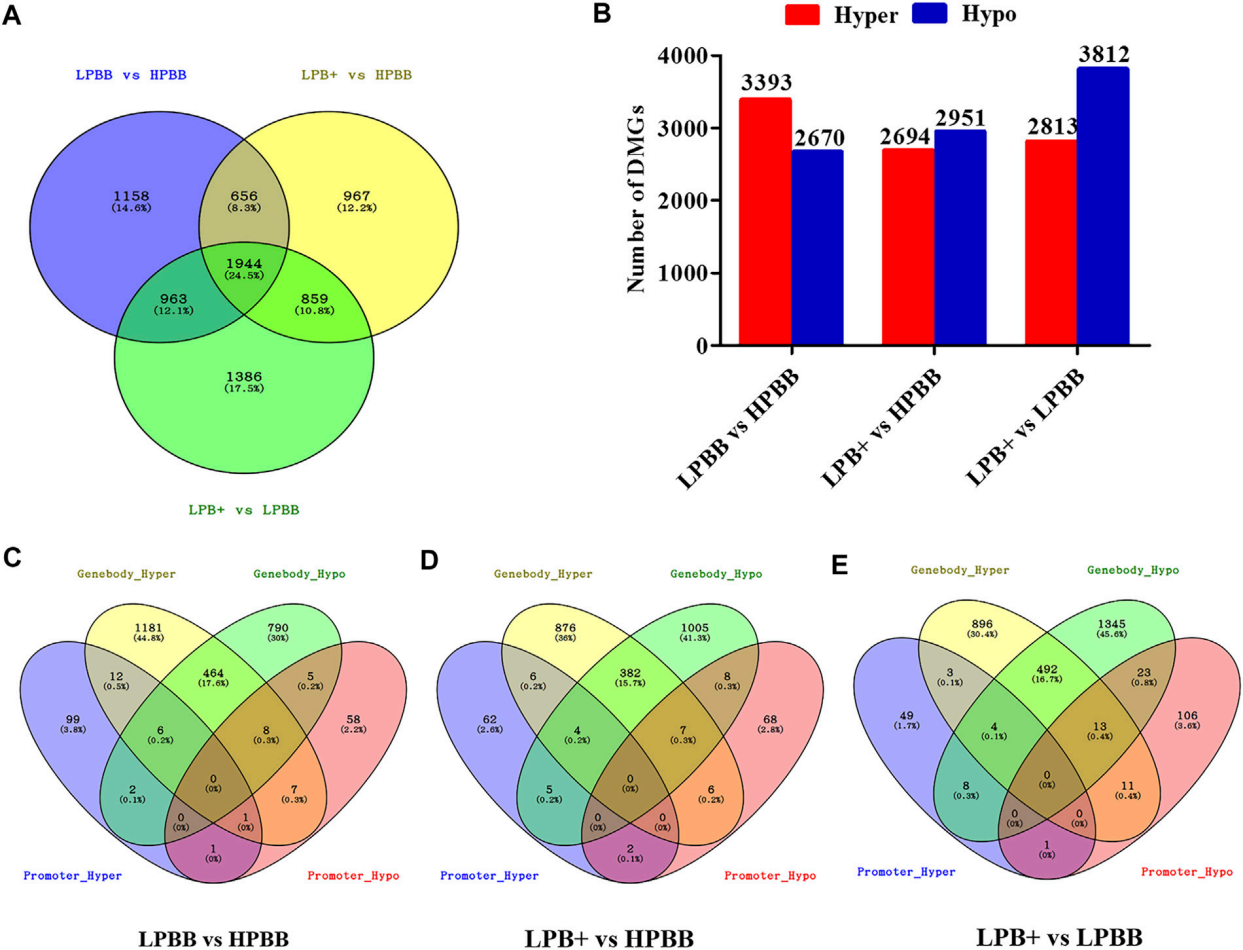
DMGs in the ovaries of different comparison groups. **(A)** DMGs that were unique or common among different comparison groups. **(B)** Number of hyper- and hypo-methylated genes in different comparison groups. **(C–E)** Venn diagram of the number of DMGs in promoter and gene body in the LPBB vs. HPBB, LPB + vs. HPBB, and LPB + vs. LPBB groups.

We identified 7,933 DMGs in the ovaries of the different comparison groups, of which 1,944 DMGs were common to all comparison groups (Figure 3A and Supplementary Table S8). Of these DMGs, 4,721 (3,393 hyper-methylated and 2,670 hypo-methylated), 4,426 (2,694 hyper-methylated and 2,951 hypo-methylated) and 5,152 (2,813 hyper-methylated and 3,812 hypo-methylated) were differentially methylated in the LPBB vs. HPBB, LPB + vs. HPBB and LPB + vs. LPBB groups, respectively (Figure 3B). Moreover, DMGs that exhibited DMRs in their promoter and gene body are shown in Figures 3C–E. The LPBB vs. HPBB, LPB + vs. HPBB and LPB + vs. LPBB groups contained 487, 404 and 528 genes, respectively, including both hyper- and hypo-methylated DMRs in their promoter and gene body.

**FIGURE 4:**
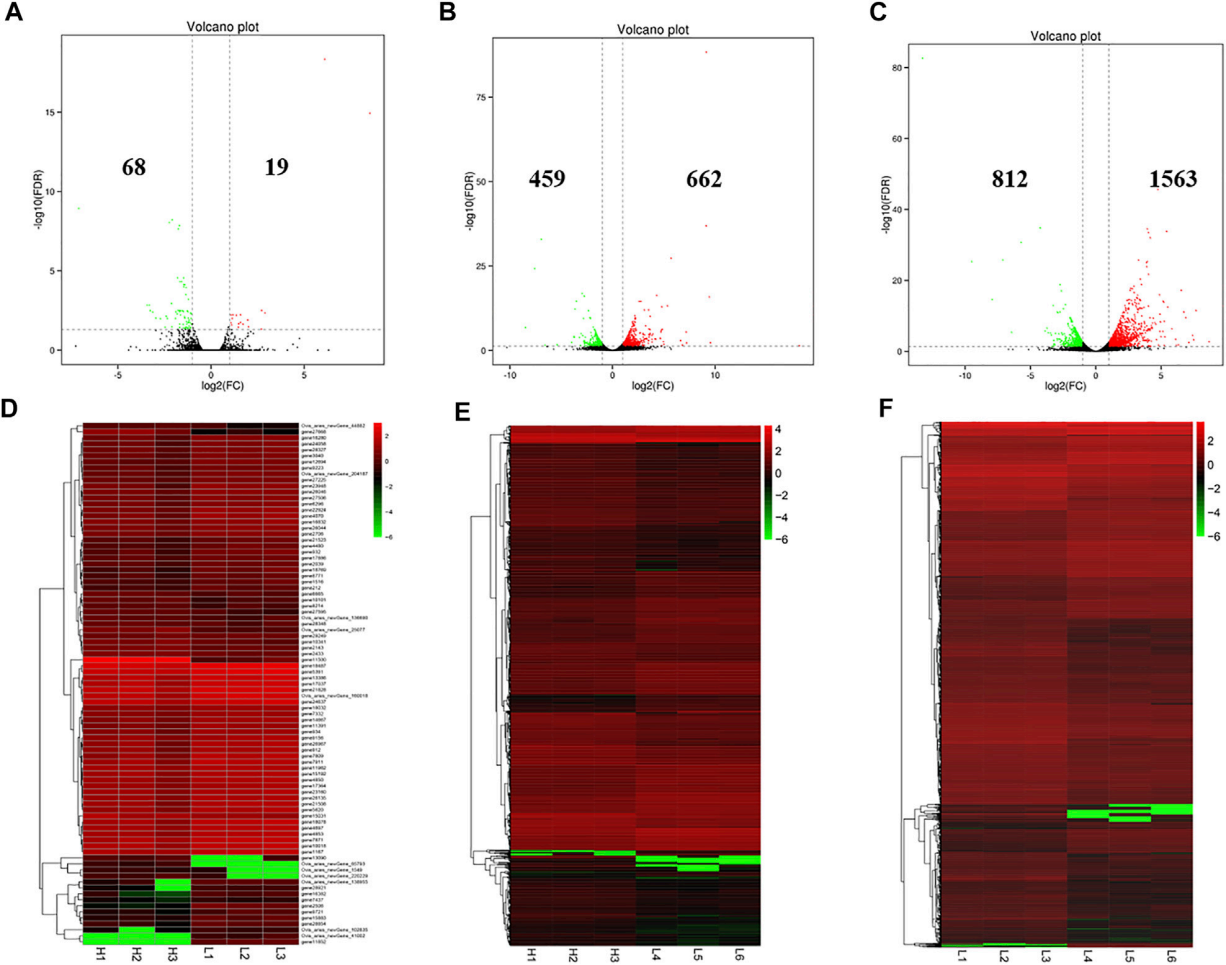
Identification of DE mRNAs. **(A–C)** Volcano plot of DE mRNAs in each group. Red indicates up-regulation and green indicates down-regulation. **(D–F)** Hierarchical clustering of DE mRNAs in each group. **(A,D)**: LPBB vs. HPBB; (B and E): LPB + vs. HPBB; and **(C,F)**: LPB + vs. LPBB groups. HPBB (H1, H2, H3); LPBB (L1, L2, L3); LPB+ (L4, L5, L6).

There were 87 (19 up-regulated and 68 down-regulated), 1,121 (662 up-regulated and 459 down-regulated) and 2,375 (1,563 up-regulated and 812 down-regulated) DE mRNAs identified in the LPBB vs. HPBB, LPB + vs. HPBB and LPB + vs. LPBB groups, respectively (Figures 4A–C). Hierarchical clustering of the DE mRNAs (Figures 4D–F) revealed the expression patterns of the individuals for each comparison.

**FIGURE 5:**
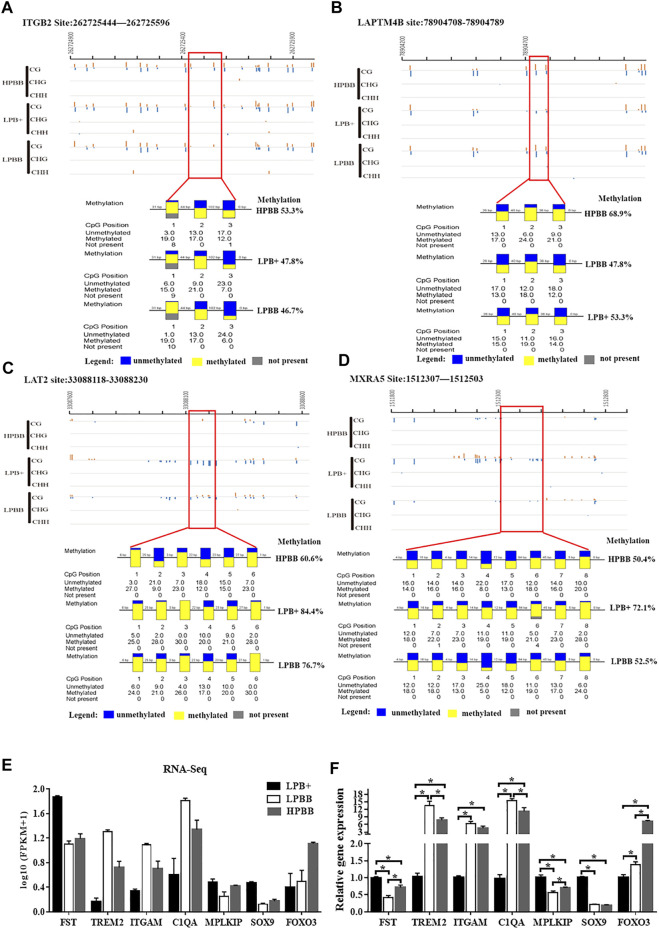
Validation of WGBS and RNA-seq data by BSP and qRT-PCR, respectively. **(A,B)** A DMR across the different groups in the promoter of the ITGB2 and LAPTM4B from 262725444 to 262725596 and from 78904708 to 78904789, respectively. **(C)** A DMR across the three groups in the intron of LAT2 from 33088118 to 33088230. **(D)** A DMR across the three groups in the distal intergenic region of MXRA5 from 1512307 to 1512503. Each box corresponds to one CpG position in the genomic sequence. Blue indicates unmethylated and yellow indicates methylated. **(E)** Seven mRNAs were randomly selected from the RNA-seq data. RNA-seq data are presented as log_10_ (FPKM+1). FPKM: Fragments Per Kilobase of transcript per Million fragments mapped. **(F)** Validation of the DEGs using qRT-PCR.

To confirm the reliability of the WGBS and RNA-seq data, four regions and seven genes were randomly selected for BSP and qRT-PCR, respectively. The results were consistent with the WGBS and RNA-seq data, suggesting that the WGBS and RNA-seq data were reliable for further study (Figure 5).

### Functional Enrichment and Interaction Network Construction

**FIGURE 6:**
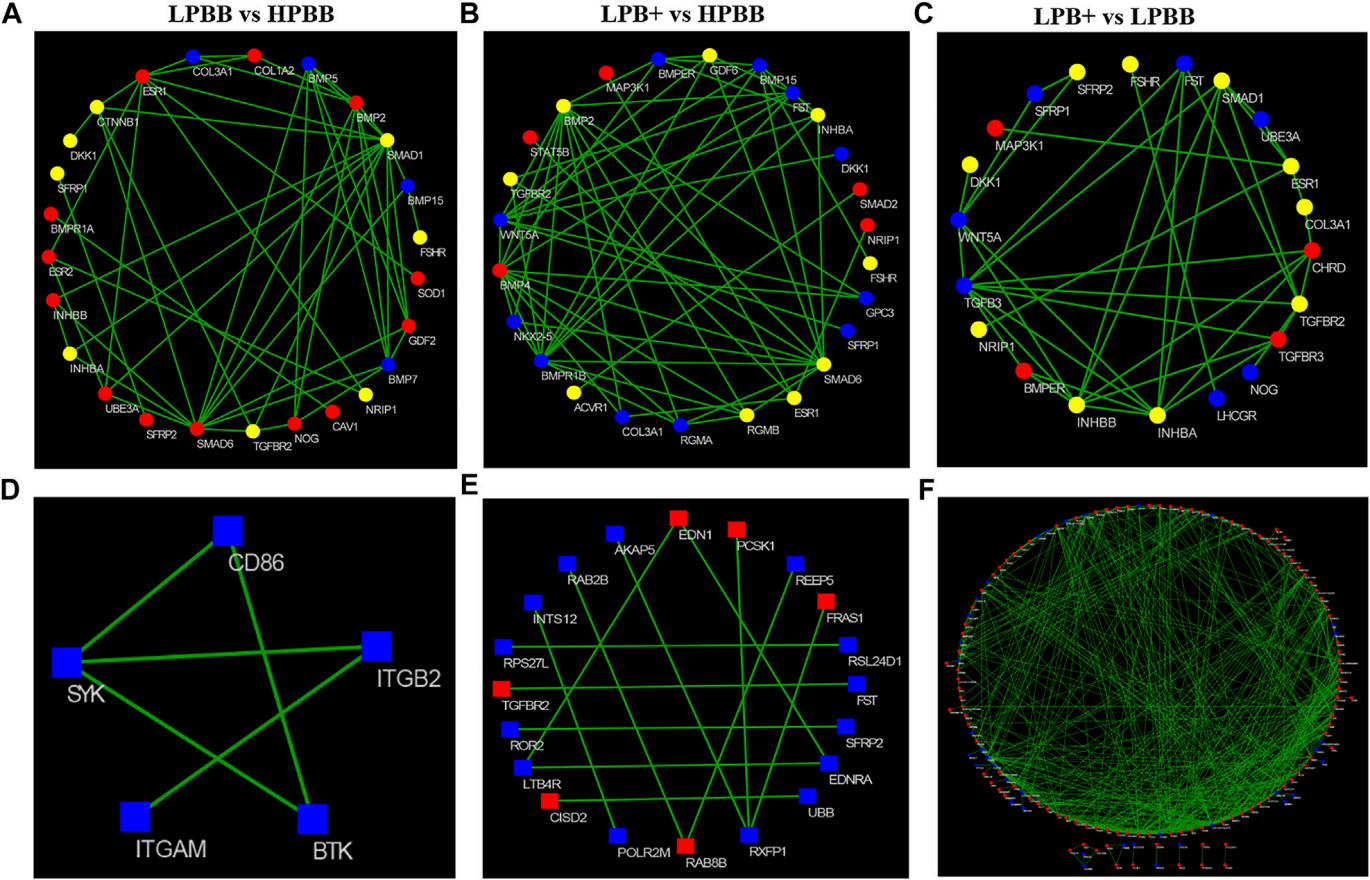
Construction of the network of DMGs and DEGs related to female reproduction. **(A–C)** Red and blue colors represent strong-hyper and strong-hypo, respectively. Yellow color represents the coexisting strong-hyper and strong-hypo. **(D–F)** Red and blue colors represent up- and down-regulated, respectively. **(A,D)** LPBB vs. HPBB; (B and E) LPB + vs. HPBB; and **(C,F)** LPB + vs. LPBB groups.

As previously mentioned, the majority of methylated cytosines were of the CG type; thus, we focused on DMGs of methylated CG for functional enrichment analysis. GO and KEGG analyses were performed to evaluate the functions of DMGs and DEGs in the ovaries of Hu sheep with different prolificacies. Across all comparisons, the DMGs were significantly enriched in the TGF-β and Wnt signaling pathways (Supplementary Figure S1). Furthermore, we selected the female reproduction associated DMGs (Supplementary Tables S9–S11) and DEGs (Supplementary Tables S12–S14) for functional enrichment and the interaction networks construction for each comparison (Figure 6). Interestingly, most of DMGs from all the comparison groups were enriched in the ovarian follicle development/rupture, BMP signaling pathway and ovulation GO terms, as well as the Wnt and TGF-β signaling pathways among all comparison groups (Supplementary Figure S2). Specifically, INHBA, TGFBR2 and SMAD7 genes of the TGF-β pathway and SFRP1, FZD1 and MAP3K7 genes of the Wnt pathway were differentially methylated among all comparison groups.

Similarly, the female reproduction associated DEGs from the LPBB vs. HPBB group were enriched in the hormone biosynthetic process and embryo implantation in GO terms (Supplementary Figure S3A), ae well as the ovarian steroidogenesis, PI3K-Akt and Rap1 signaling pathways (Supplementary Figure S3B). Meanwhile, female reproduction associated DEGs from both the LPB + vs. HPBB and LPB + vs. LPBB groups were enriched in the female gonad and ovarian follicle development GO terms (Supplementary Figures S3C,E), as well as the ovarian steroidogenesis, PI3K-Akt, TGF-β and Wnt signaling pathways (Supplementary Figures S3D,F).

### Correlation Analysis Between DNA Methylation and Gene Expression

**FIGURE 7:**
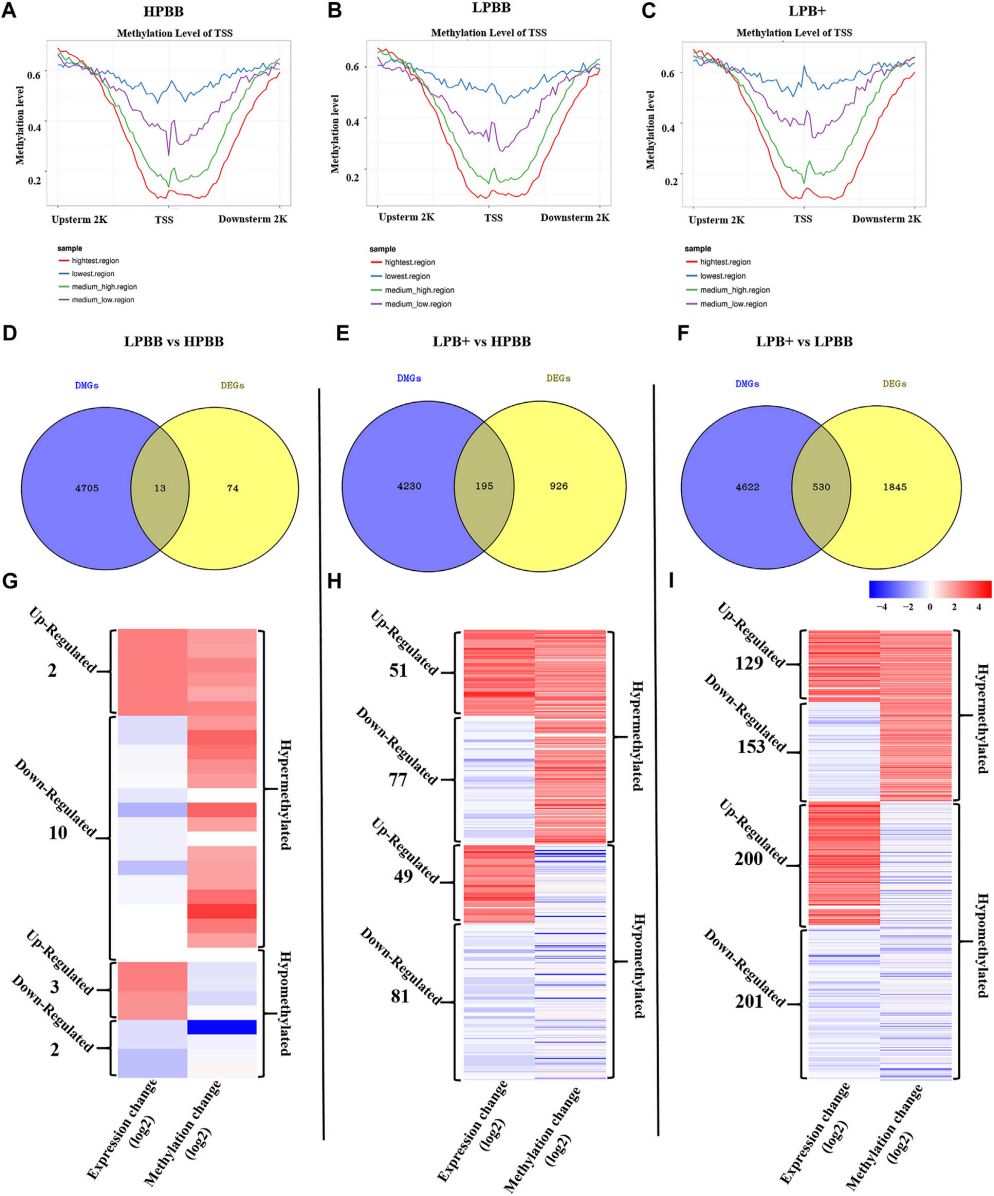
Integrative analysis of the DNA methylome and transcriptome in the ovaries of different groups. **(A–C)** DNA methylation level distribution around TSS of four levels of gene expression in the different groups. **(D–F)** Venn diagram shows the common DMGs and DEGs. **(G–I)** Heatmap showing the differentially methylated levels and corresponding genes change in the different comparison groups.

As shown in Figures 7A–C, a significant negative correlation was observed between DNA methylation level around the TSS and gene expression. In the LPBB vs. HPBB, LPB + vs. HPBB and LPB + vs. LPBB groups, we found that 13, 195 and 530 of the DMGs, respectively, associated with DEGs (Figures 7D–F and Supplementary Tables S15–S17). Moreover, the heatmap was constructed to visualize the relationship between DMGs and DEGs in the different comparison groups (Figures 7G–I). Many DMGs contained more than one DMR. For example, five DMRs in NDST4 were hyper-methylated and one DMR in NDST4 was hypo-methylated in the HPBB group compared to that in the LPBB group. In the LPBB vs. HPBB group, we identified 10 hyper-methylated genes with down-regulated expression levels in the HPBB group, including genes related to the Hippo (ITGB2), PI3K-Akt (SYK) and Rap1 (ITGB2) signaling pathways, while three genes were hypo-methylated and up-regulated in the HPBB group compared to that in the LPBB group (Figure 7G and Supplementary Table S15). In the LPB + vs. HPBB group, 77 genes were hyper-methylated and down-regulated in the HPBB group, and these genes were related to the regulation of the TGF-β (ID2), estrogen (LOC101114987) and oocyte meiosis (LOC101112318 and PTTG1) signaling pathways. In contrast, 49 genes were hyper-methylated and down-regulated in the LPB + group, and these genes were related to the TGF-β (TGFBR2), PI3K-Akt (COL4A1, LOC101115805 and COL24A1), FoxO (TGFBR2 and FOXO3) and insulin (CBLB, PDE3A and RIMS2) signaling pathways (Figure 7H and Supplementary Table S16). In the LPB + vs. LPBB group, 153 genes were hyper-methylated and down-regulated in the LPBB group, and these genes were related to the PI3K-Akt (PRKAA2, FGF10, FGF12 and LOC101103187), TGF-β (ID2) and ovarian steroidogenesis (FSHR) pathways. We found that 201 genes were hypo-methylated and up-regulated in the LPBB group compared to that in the LPB + group, and these genes were related to the PI3K-Akt (COL24A1, TLR4, SYK, ITGAV, ITGA7, VWF, RELN, FLT4, KIT, SGK1, THBS2, COL4A1 and LOC101115805), TGF-β (CDKN2B and TGFBR2) and ovarian steroidogenesis (STAR and PRKX) pathways (Figure 7I and Supplementary Table S17). In addition, some genes exhibited coinciding methylation and expression patterns in three comparison groups (Figures 7G–I and Supplementary Tables S15–S17). Interestingly, we found that ITGB2 and LAPTM4B genes, which were hyper-methylated (DMRs in their promoter) and down-regulated in LPBB vs. HPBB group and both LPB + vs. HPBB and LPB + vs. LPBB group, respectively, to confirm the negative correlation between DNA methylation and gene expression.

### Integrative Analysis of ITGB2 and LAPTM4B Methylation and Expression

**FIGURE 8:**
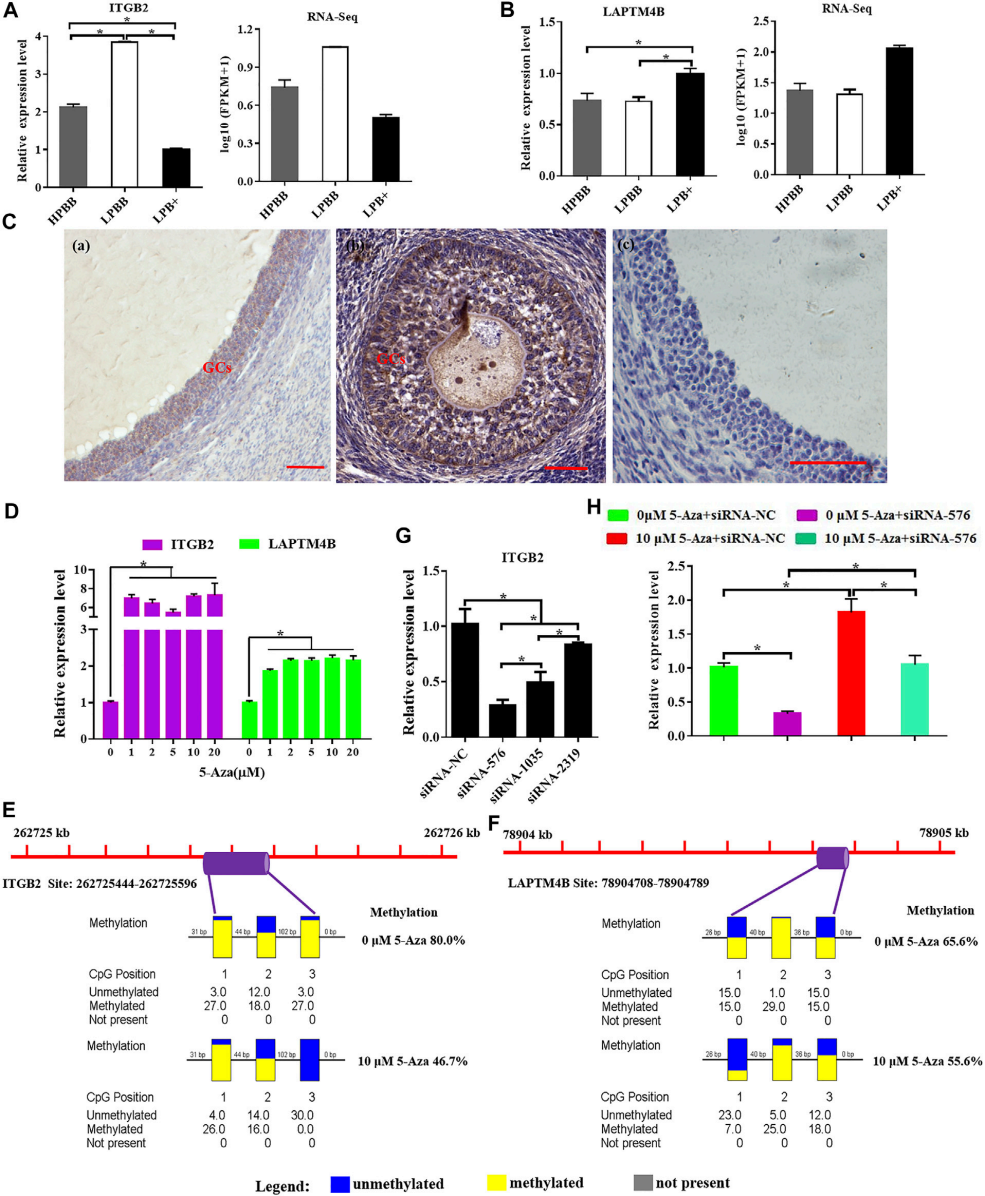
Integrative analysis of methylation and expression levels of ITGB2 and LAPTM4B. **(A,B)** ITGB2 and LAPTM4B mRNA expression in the ovaries were detected by qRT-PCR. **(C)** Localization of LAPTM4B in the ovaries using immunohistochemistry. Scale bars = 50 μm. **(D)** ITGB2 and LAPTM4B mRNA expression levels in cultured GCs were detected after treatment with various concentrations (0, 1, 2, 5, 10, and 20 μM) of 5-Aza treatment. **(E,F)** Methylation levels of ITGB2 and LAPTM4B in cultured GCs after 10 μM 5-Aza treatment. **(G)** Suppression efficiency of ITGB2 was evaluated by qRT-PCR. **(H)** mRNA level of ITGB2 in cultured GCs stimulated by 5-Aza with or without siRNA-ITGB2.

ITGB2 mRNA expression level in the LPBB group was significantly higher than that in the LPB+ and HPBB groups; with the expression being higher in the HPBB group than in the LPB + group (Figure 8A). LAPTM4B mRNA expression level in the LPB + group was significantly higher than that in the LPBB and HPBB groups (Figure 8B). These results were consistent with the RNA-seq data. Meanwhile, LAPTM4B protein was predominantly localized in the GCs of the antral follicle (Figure 8C).

Next, we evaluated the effect of 5-Aza on ITGB2 and LAPTM4B expression in cultured GCs, and the results showed that ITGB2 and LAPTM4B mRNA expression were significantly higher in the 5-Aza treatment group (Figure 8D). Moreover, following the treatment of GCs with 5-Aza, the methylation level of the LAPTM4B and ITGB2 promoter decreased compared to that of the control cells (Figures 8E,F). Additionally, the mRNA expression of ITGB2 was significantly decreased using siRNA-specific ITGB2 (Figure 8G), subsequently, we demonstrated that the treatment of 5-Aza inhibited ITGB2-specific siRNA-reduced ITGB2 expression in GCs (Figure 8H).

## Discussion

Fecundity is an economically important trait in the sheep industry. Therefore, investigating the molecular mechanisms of sheep fecundity may assist in accelerating the breeding process. Herein, we systematically investigated the genome-wide DNA methylation and gene expression profiles in the ovaries of Hu sheep with different prolificacies and genotypes by WGBS and RNA-seq, respectively. Although it has been demonstrated that the homozygous mutation (BB) had the higher fecundity than the heterozygous mutation (B+) or wild-type (++), significant differences in prolificacy phenotypes were found in the same genotype (BB) of Hu sheep with under the same feeding conditions. Therefore, we conducted an integrated analysis of DNA methylation and gene expression patterns was performed to reveal the manner in which DNA methylation may regulate prolificacy by affecting gene expression.

WGBS revealed that approximately 3.58% of cytosine sites were methylated in the ovaries of Hu sheep, with the highest proportion of CG methylation context. This finding is corroborated by previous reports in other animals (Yuan et al., 2016; Hwang et al., 2017; Fan et al., 2020). Moreover, the DNA methylation of the CG context from the ovaries of Hu sheep exhibited significant hyper-methylation levels (90–100%), but CHH and CHG exhibited hypo-methylation levels (10–30%), which is consistent with the results of a previous study in the ovaries of pigs (Yuan et al., 2016). These results suggest that the CG methylation was high-efficiently maintained in the ovaries of Hu sheep.

The majority of DMRs in the ovaries of Hu sheep exhibit a similar distribution to that observed in the ovaries or muscle of pigs and Hu sheep (Hwang et al., 2017; Yang et al., 2017; Fan et al., 2020), with the mainly located at the intergenic and intron elements. Accordingly, we identified numerous DMGs among the three comparison groups. As described above, focusing on the CG methylation context of DMGs, and we found that the common DMGs were significantly enriched in the TGF-β and Wnt signaling pathways, which have been confirmed to be involved in fecundity (Chu et al., 2007; Gao et al., 2020). For example, INHBA is associated with follicle development (Cui et al., 2020; Li et al., 2021), oocyte maturation (Tesfaye et al., 2009) and fecundity (Hiendleder et al., 1996; Yu et al., 2019). In the present study, INHBA was found to contain five distal intergenic DMRs, with four DMRs being hyper-methylated and one being hypo-methylated in the LPB + vs. HPBB group. Moreover, six DMRs (five distal intergenic and one intron) were identified in INHBA, of which five were hypo-methylated and one was hyper-methylated in the LPB + vs. LPBB group. FZD1 has been confirmed to regulate certain biological processes, including oocyte maturation, female fertility (Lapointe et al., 2012), embryonic development (Tribulo et al., 2017) and ovary development (Tepekoy et al., 2019). The methylation level of the 3′-UTR of FZD1 in the HPBB group was higher than that in the LPB + group. In addition, most DMGs were enriched in the ovulation and ovarian follicle development biological processes. These results supported the hypothesis that DNA methylation as a regulator of epigenetic modification could influence the prolificacy phenotype (Hwang et al., 2017; Miao et al., 2017; Zhang et al., 2017).

To understand how DNA methylation influences the prolificacy of Hu sheep, here, for the first time, we systematically analyzed and compared the genome-wide DNA methylation and transcriptome of ovaries from Hu sheep with different prolificacies and genotypes. The number of DMGs-DEGs in the LPBB vs. HPBB group was lower than that in both the LPB + vs. HPBB and LPB + vs. LPBB groups. This finding suggests that, although changes in DNA methylation may influence fecundity, the differences are mainly attributed to the genotype. The most significant results of this study showed that an inverse relationship was observed between DNA methylation and gene expression in the HPBB, LPBB, and LPB + groups, which is in agreement with the results of previous studies (Jadhao et al., 2017; Wang et al., 2017; Yang et al., 2017).

DNA methylation status in the promoter and gene body regions regulates gene expression by changing transcription efficiency or chromatin structure (Jones 2012; Yang et al., 2014). Therefore, we further selected the genes (ITGB2 and LAPTM4B) that exhibited inverse changes in DNA methylation and gene expression, and those with DMRs in the promoter or gene body for subsequent analyses. For example, the promoter region of ITGB2, an important regulator of embryo implantation (Guo et al., 2018), oocyte maturation (Antosik et al., 2016) and follicle development (Kisliouk et al., 2007; Antosik et al., 2016), was hyper-methylated in the HPBB group compared to that in the LPBB group; however, its expression was higher in the LPBB group than in the HPBB group. Meanwhile, ITGB2 expression level in atretic follicles was higher than in healthy follicles (Kisliouk et al., 2007). Up-regulated expression of ITGB2 in the LPBB group may be responsible for the increased number of atretic follicles. Previous studies have reported that LAPTM4B is expressed in the reproductive organs and reproductive diseases in bovine or human (Yang et al., 2008; Ndiaye et al., 2015; Meng et al., 2016). Ndiaye et al. (2015) reported that LAPTM4B expression was higher in large dominant follicles than in small antral follicles. The results of the present study showed that the LAPTM4B promoter was hyper-methylated and LAPTM4B expression was down-regulated in the HPBB group compared to that in the LPB + group. Moreover, the relationship between ITGB2 and LPATM4B expression and epigenetic modifications in the ovaries and cultured GCs were assessed, and the results showed an inverse relationships occurred for both. These findings may, to some extent, explain the significant differences in phenotypic variation among Hu sheep.

In summary, the present study systematically integrated DNA methylation and gene expression profiles in the ovaries of Hu sheep with different phenotypes and genotypes, indicating the potential mechanisms underlying on prolificacy phenotypic variation and providing new insights into the genetic mechanism responsible for the excellent fecundity of Hu sheep. As several factors, including physiological, environmental and diet, have been shown to affect phenotypic variation (Peaston and Whitelaw, 2006). Therefore, further studies are needed to fully understand the effects of epigenetic modification on the fecundity of Hu sheep, which may contribute to better reproductive efficiency.

## Data Availability

The raw data of transcriptome and DNA methylome have been deposited into the Sequence Read Archive database (https://www.ncbi.nlm.nih.gov/sra) under accession numbers are PRJNA681364 and PRJNA758179, respectively.

## Supplementary Material

Supplementary Figure S1 KEGG pathway enrichment analysis of DMGs. **(A)** LPBB vs. HPBB, **(B)** LPB+ vs. HPBB, and **(C)** LPB+ vs. LPBB groups.

Supplementary Figure S2 GO and KEGG enrichment analysis of DMGs related to female reproduction. **(A)** LPBB vs. HPBB, **(B)** LPB+ vs. HPBB, and **(C)** LPB+ vs. LPBB groups.

Supplementary Figure S3 GO and KEGG pathway enrichment analysis of DEGs related to female reproduction. **(A,B)** LPBB vs. HPBB, **(C,D)** LPB+ vs. HPBB, and **(E,F)** LPB+ vs. LPBB groups.
