# Supplementary figures and images for: Integrative Genome-Wide DNA Methylome and Transcriptome Analysis of Ovaries from Hu Sheep with High and Low Prolific
Source: Front Cell Dev Biol. 2022 Feb 3;10:820558. doi: 10.3389/fcell.2022.820558 (PMC8850840; doi:10.3389/fcell.2022.820558)

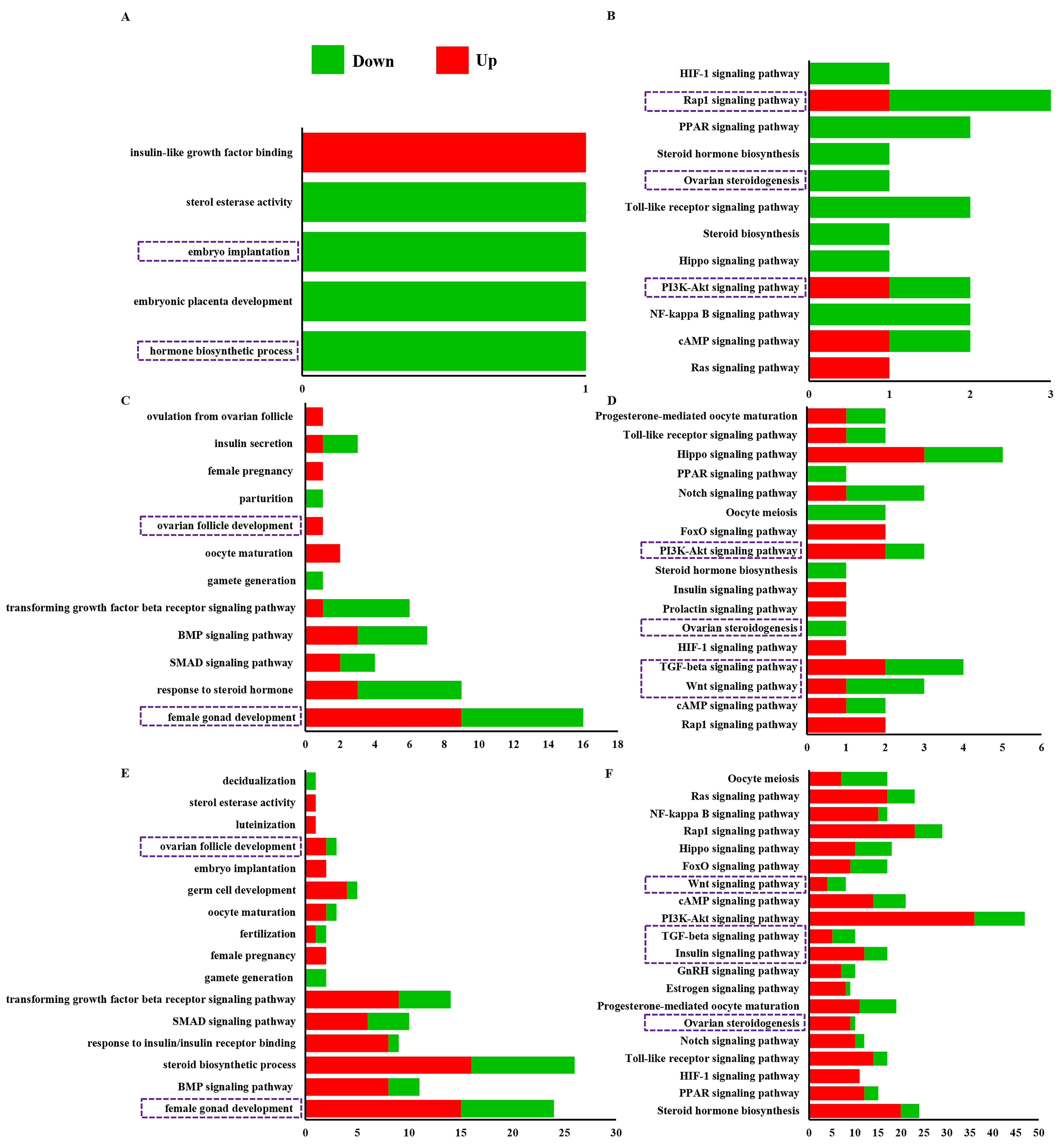

Supplement: Supplementary file 1 [file Image3.TIF]

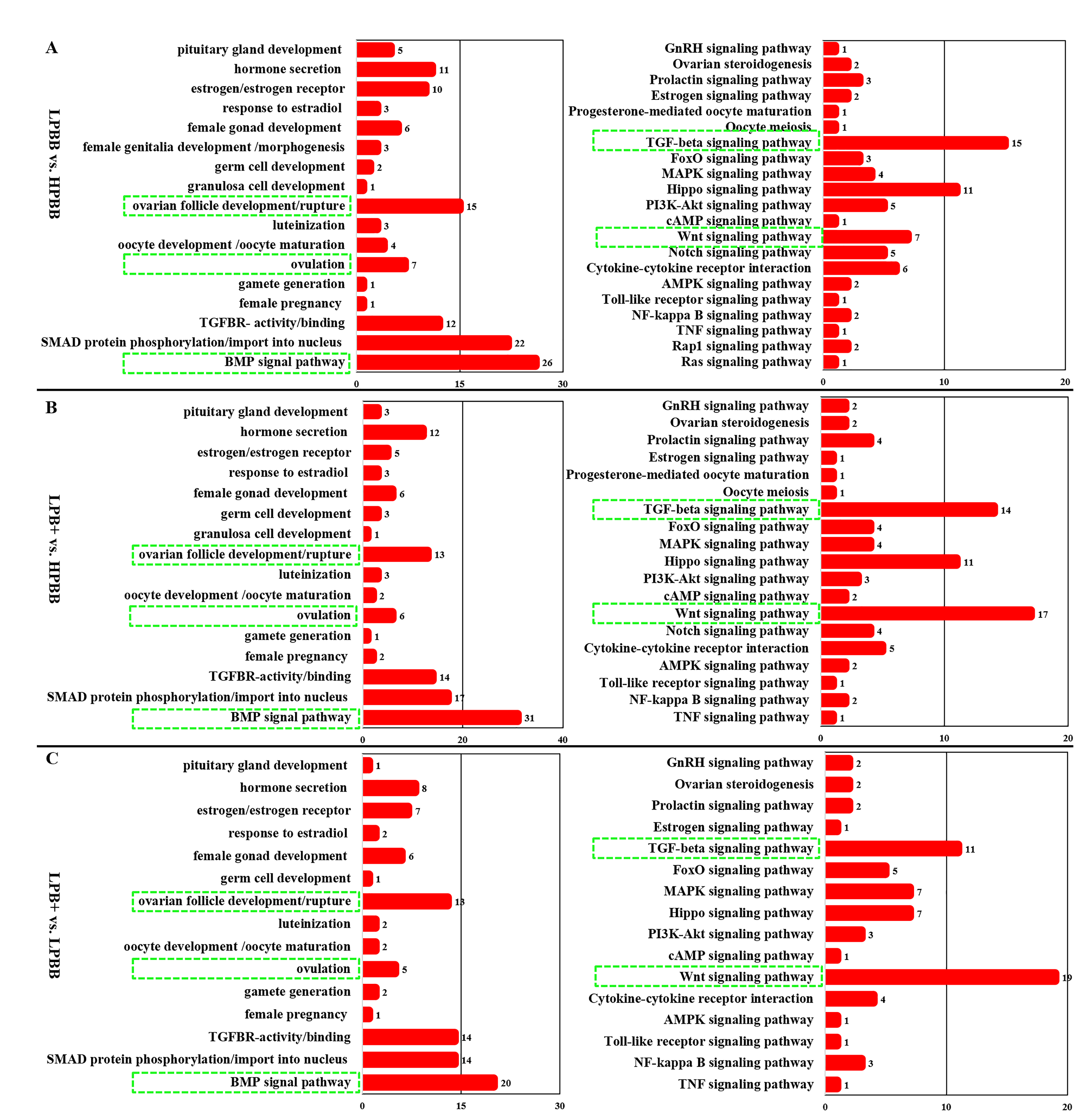

Supplement: Supplementary file 3 [file Image2.TIF]

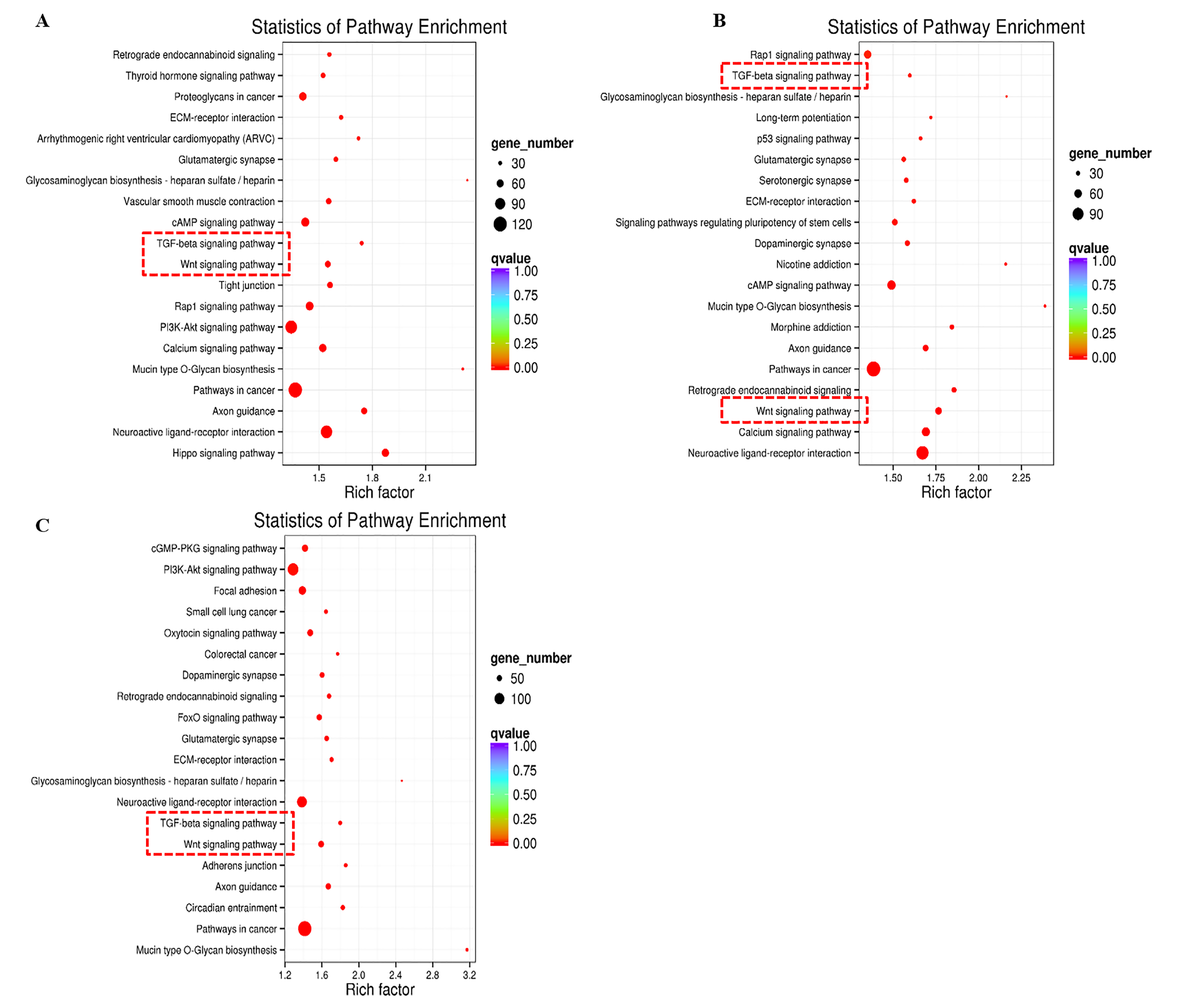

Supplement: Supplementary file 4 [file Image1.TIF]
